# Candida glabrata Yap6 Recruits Med2 To Alter Glycerophospholipid Composition and Develop Acid pH Stress Resistance

**DOI:** 10.1128/AEM.01915-20

**Published:** 2020-11-24

**Authors:** Pei Zhou, Xiaoke Yuan, Hui Liu, Yanli Qi, Xiulai Chen, Liming Liu

**Affiliations:** a State Key Laboratory of Food Science and Technology, Jiangnan University, Wuxi, Jiangsu, China; b Key Laboratory of Industrial Biotechnology, Ministry of Education, Jiangnan University, Wuxi, Jiangsu, China; c International Joint Laboratory on Food Safety, Jiangnan University, Wuxi, Jiangsu, China; d School of Biotechnology, Jiangnan University, Wuxi, Jiangsu, China; University of Illinois at Urbana-Champaign

**Keywords:** *Candida glabrata*, Mediator subunit Med2, glycerophospholipid, low-pH stress, transcriptome

## Abstract

Candida glabrata is a high-performance microbial cell factory for the production of organic acids. To elucidate the role of the C. glabrata Mediator tail subunit Med2 (*Cg*Med2) at pH 2.0, we deleted or overexpressed *Cg*Med2 and used transcriptome analysis to identify genes that are regulated by *Cg*Med2. At pH 2.0, the deletion of *Cg*Med2 resulted in a cell growth decrease of 26.1% and a survival decrease of 32.3%. Overexpression of *Cg*Med2 increased cell growth by 12.4% and cell survival by 5.9% compared to the wild-type strain. Transcriptome and phenotypic analyses identified *Cg*Yap6 as a transcription factor involved in acid pH stress tolerance. Deletion of *Cg*Yap6 caused growth defects, whereas its overexpression enhanced cell growth at pH 2.0. Furthermore, total glycerophospholipid content and membrane integrity decreased by 33.4% and 21.8%, respectively, in the *Cg*Med2Δ strain; however, overexpression of *Cg*Med2 increased the total glycerophospholipid content and membrane integrity by 24.7% and 12.1%, respectively, compared with those of the wild-type strain at pH 2.0. These results demonstrated that under acid pH stress, *Cg*Med2 physically interacts with *Cg*Yap6, which translocates from the cytoplasm to the nucleus after being phosphorylated by the protein kinase *Cg*Yak1. Once in the nucleus, *Cg*Yap6 recruits *Cg*Med2 to express glycerophospholipid-related genes. Our study elucidated the function of *Cg*Med2 under acid pH stress and provides a potential strategy to equip Candida glabrata with low-pH resistance during organic acid fermentation.

**IMPORTANCE** This study investigated the function of the Mediator tail subunit *Cg*Med2 in C. glabrata under low-pH stress. The protein kinase *Cg*Yak1 activates *Cg*Yap6 for the recruitment of *Cg*Med2, which in turn increases glycerophospholipid content and membrane integrity to confer low-pH stress tolerance. This study establishes a new link between the Mediator tail subunit and transcription factors. Overall, these findings indicate that *Cg*Med2 is a novel target to induce the low-pH stress response in C. glabrata.

## Abstract (precis)

This study investigated the function of the Mediator tail subunit *Cg*Med2 in C. glabrata under low-pH stress. The protein kinase *Cg*Yak1 activates *Cg*Yap6 for the recruitment of *Cg*Med2, which in turn increases glycerophospholipid content and membrane integrity to confer low-pH stress tolerance. This study establishes a new link between the Mediator tail subunit and transcription factors. Overall, these findings indicate that *Cg*Med2 is a novel target to induce the low-pH stress response in C. glabrata.

## INTRODUCTION

Organic acids have become increasingly important in biotechnology, with main applications in the food, pharmaceutical, and textile industries (1). Among the several different microorganisms used to produce organic acids, Candida glabrata is a high-performance yeast used to produce malic acid (2), fumaric acid (3), and α-ketoglutaric acid (4). However, during the production, the accumulation of extracellular acids considerably decreases the pH of the fermentation broth, thereby inhibiting cell growth and ultimately reducing the production of the target acid (5). Although exogenous addition of alkaline reagents, such as NaOH and CaCO_3_, helps stabilize the correct working pH, it increases the osmotic pressure and the cost of downstream separation and purification (6). Therefore, it is urgent to find efficient strategies to improve the tolerance of C. glabrata to complex industrial environmental conditions.

Many researchers have proposed different methods to increase yeast tolerance to acid stress, such as adaptive laboratory evolution (ALE), transporter engineering, transcription factor engineering, modification of specific genes, and Mediator complex engineering (7, 8). ALE strategies have been proven effective in the production of organic acid (9, 10). For example, ALE has been used to explore the tolerance mechanisms of Saccharomyces cerevisiae in the presence of inhibiting concentrations of dicarboxylic acids. Moreover, reverse metabolic engineering amplification of Qdr3 (a transporter associated with multidrug resistance) conferred tolerance to dicarboxylic acids while enhancing the production of muconic acid in engineered S. cerevisiae (10). Furthermore, transporter engineering has been used to manipulate the expression level of proton pumps, including plasma membrane H^+^ antiporter Aqr1, proton pump Pma1, and vacuolar proton pumping Pep3, thereby improving acid stress tolerance in yeast (11–13). Transcription factors, such as Haa1, Msn2, Asg1, and Hal9, were found to be involved in the response to acid stress (14–16). For example, under acidic conditions, Asg1 regulates the expression of several genes related to the plasma membrane, cell wall organization, mitogen-activated protein kinase (MAPK) signaling pathway, and trehalose accumulation (14). Moreover, modification of specific genes increases the tolerance to acid stress in yeast (17, 18). For example, deletion of Atg22 in S. cerevisiae resulted in morphology changes that enhanced cell protection against acidic environments and increased the production of intracellular amino acids to respond to amino acid starvation (18). To resist environmental stresses, cells require alteration of the expression level of several different genes, which is difficult to achieve through continuous multigene modification (19). However, the Mediator complex can be engineered to reprogram the gene expression network to improve cell tolerance to low-pH stress (20, 21). For example, overexpression of *Cg*Med3 increases the expression of genes related to lipid metabolism, enhancing the levels of C_18:0_, C_18:1_, and ergosterol and, consequently, membrane integrity and pyruvate production (20). Therefore, Mediator complex engineering offers a potential strategy to increase acid tolerance and organic acid production in C. glabrata.

The Mediator complex is an evolutionarily conserved, multiprotein complex required for RNA polymerase II-driven transcription; transcription factors recruit Mediator in the upstream activation sequence of the target gene to regulate gene function (22). In S. cerevisiae, the Mediator complex consists of 25 subunits and is organized in four modules designated head, middle, tail, and kinase (23). The tail module comprises five subunits: Med2, Med3, Med5, Med15, and Med16 (24). The main function of the tail module is to integrate the transcriptional regulatory signals from sequence-specific transcription factors (24, 25). Therefore, the tail module can respond to a variety of physiological processes (26). For example, in C. glabrata, Med3 regulates cell growth by coordinating the homeostasis of cellular acetyl coenzyme A (acetyl-CoA) metabolism and the cell cycle cyclin Cln3 (27). Adaptive evolution and integrated systems biology studies on S. cerevisiae revealed that, in addition to abolishing the Crabtree effect (i.e., the ability to rapidly consume glucose and produce ethanol with antiseptic properties), Med2 mutation caused global redistribution of yeast cell metabolism (28). Moreover, the tail module is involved in the response to several environmental stresses (29). In C. glabrata, Med3 and Med15 regulate the expression of genes related to lipid metabolism, specifically increasing the rigidity of the cell membrane and improving the viability of the yeast under acid stress (20, 21). Furthermore, in *Candida* spp., Med2 not only regulates cell viability under diverse stress conditions but also facilitates filamentous growth (30). However, the mechanism by which Med2 controls cell survival in adverse environment remains unclear.

Here, we used transcriptome sequencing and metabolomics to elucidate the role of C. glabrata Med2 (*Cg*Med2) in low-pH perturbation response. We discovered that the protein kinase *Cg*Yak1 activates the transcription factor *Cg*Yap6 inside the nucleus by recruiting the mediator *Cg*Med2, which activates glycerophospholipid genes that resist low-pH stress. This study provides insight into cellular reprogramming in response to low pH and establishes a regulatory circuitry among *Cg*Med2, *Cg*Yak1, and *Cg*Yap6.

## RESULTS

### *Cg*Med2 facilitates yeast growth at pH 2.0.

**FIG 1:**
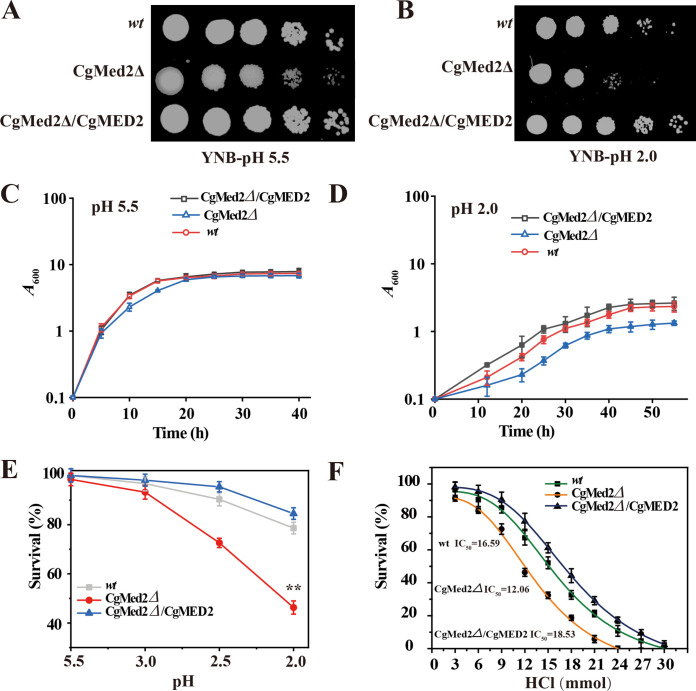
*Cg*Med2 is essential for cell growth under low-pH stress. (A and B) The wild-type (wt), *Cg*Med2Δ, and *Cg*Med2Δ/*Cg*MED2 strains were spotted on YNB plates at pH 5.5 and pH 2.0. (C and D) Growth curves of the wild-type (wt), *Cg*Med2Δ, and *Cg*Med2Δ/*Cg*MED2 at pH 5.5 and pH 2.0. (E) Cell survival of all three strains at different pHs. (F) IC_50_s of the wild-type (wt), *Cg*Med2Δ, and *Cg*Med2Δ/*Cg*MED2 strains at different concentration of HCl. Error bars indicate standard deviations. *, *P* < 0.05; **, *P* < 0.01; ***, *P* < 0.001 (compared to the corresponding wild-type strain, as determined by a *t* test).

To investigate whether *Cg*Med2 was necessary for C. glabrata growth at pH 2.0, the wild-type, *Cg*Med2Δ, and *Cg*Med2Δ*/Cg*MED2 (the MED2 overexpression strain was constructed with plasmid pY26, and the level of CgMed2 overexpression was 4.3-fold than that of wild-type strain as determined by qRT-PCR [see Fig. S1 in the supplemental material]) strains were spotted and grown on yeast nitrogen base (YNB) plates at pH 5.5 and pH 2.0. The deletion of *Cg*Med2 caused significant growth decline at pH 2.0, whereas its overexpression enhanced growth compared to that of the wild-type strain (Fig. 1A and B). Comparison of the growth curves of the three strains at pH 5.5 and pH 2.0, showed that the final biomass of *Cg*Med2Δ decreased by 7.58% compared to that of the wild-type strain at pH 5.5. At pH 2.0, the final biomass of the *Cg*Med2Δ and *Cg*Med2Δ*/Cg*MED2 strains was 26.1% lower and 12.4% higher, respectively, than that of the wild-type strain (Fig. 1C and D). Furthermore, at pH 2.0, the wild-type, *Cg*Med2Δ, and *Cg*Med2Δ/*Cg*MED2 cell survival was 78.6%, 46.3%, and 84.5%, respectively (Fig. 1E). Finally, the half-maximal inhibitory concentration (IC_50_) values for the wild-type, *Cg*Med2Δ, and *Cg*Med2Δ*/Cg*MED2 strains were 16.59, 12.06, and 18.53 mM HCl, respectively (Fig. 1F). These results suggest that CgMed2 plays an important role in the growth of C. glabrata at pH 2.0.

### Global transcriptome analysis of the wild-type and *Cg*Med2Δ strains at pH 2.0.

**FIG 2:**
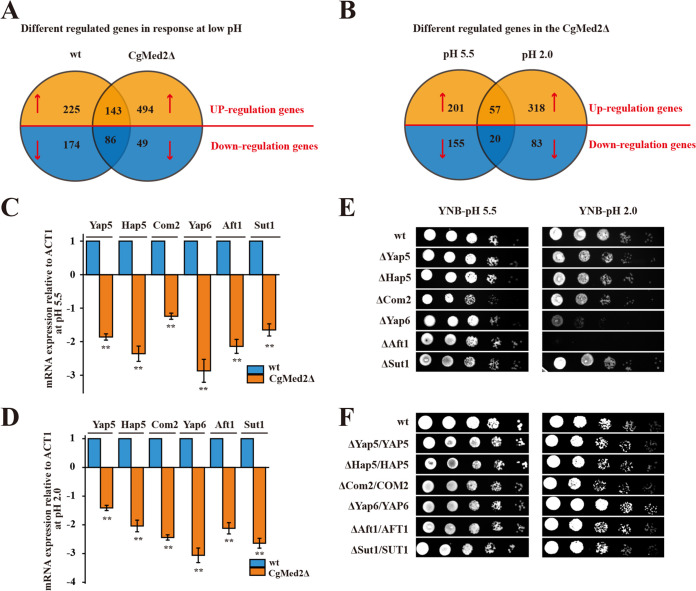
Global transcriptome analysis of the mutant *Cg*Med2Δ and the wild-type strain. (A) Venn diagrams depicting the numbers of upregulated and downregulated genes in the wild-type strain and *Cg*Med2Δ strain under the pH 2.0 condition compared with the expression levels of those genes in the corresponding strains under the pH 5.5 condition. (B) Venn diagrams depicting the numbers of upregulated and downregulated genes in the *Cg*Med2Δ strain under pH 5.5 and pH 2.0 conditions compared with the expression levels of those genes in the wild-type strains under pH 5.5 and pH 2.0 conditions. (C and D) Quantitative reverse transcription-PCR (qRT-PCR) verified the mRNA expression levels of the most downregulated transcription factor genes, calculated relative to the *ACT1* level, at pH 2.0 and pH 5.5. Error bars indicate the standard deviations. *, *P* < 0.05; **, *P* < 0.01; ***, *P* < 0.001 (compared to the corresponding wild-type strain, as determined by a *t* test). (E) The most downregulated transcription factor genes were deleted, and the mutant strains were spotted on YNB plates under pH 2.0 and pH 5.5 conditions. (F) The most downregulated transcription factor genes were overexpressed, and the mutant strains were spotted on YNB plates under pH 2.0 and pH 5.5 conditions.

To further describe the role of *Cg*Med2 in C. glabrata, transcriptome sequencing (RNA-seq) was conducted to compare global gene expression in the wild-type and *Cg*Med2Δ strains at pH 5.5 and 2.0. Restrictive thresholds [|log_2_(fold change)| ≥ 2.0; false-discovery rate (FDR) < 0.05] of significantly expressed genes were used for screening. First, the differentially expressed genes (DEGs) between strains grown at pH 2.0 and the normal condition (pH 5.5) were identified in both the wild-type and *Cg*Med2Δ mutant strains (Fig. 2A). Transcriptional profiling and Kyoto Encyclopedia of Genes and Genomes (KEGG) analysis revealed that signal transduction and amino acid metabolism were the pathways most significantly affected by low pH in the wild-type strain, whereas signal transduction and transport were the most significantly affected pathways in *Cg*Med2Δ mutant strains. In addition, 299 DEGs were common between wild-type and *Cg*Med2Δ mutant strains, including 143 upregulated and 86 downregulated genes. The commonly upregulated genes were involved in amino acid metabolism, lipid metabolism, energy metabolism, and cofactor metabolism, whereas the commonly downregulated genes were enriched in KEGG processes such as carbohydrate metabolism, meiosis, and transport.

We next directly compared the DEGs between the *Cg*Med2Δ mutant and wild-type strains at each pH condition (Fig. 2B). At pH 5.5, DEGs between the strains were involved mainly in carbohydrate metabolism and translation; however, at pH 2.0, the DEGs were involved in signal transduction and catabolism. A total of 77 common DEGs were identified between the two pH conditions, including 57 upregulated and 20 downregulated genes. KEGG analysis showed that the commonly upregulated genes were involved in glycan metabolism, amino acid metabolism, lipid metabolism, and DNA repair, whereas the commonly downregulated genes were enriched in processes such as membrane transport, protein folding and degradation, and meiosis.

To investigate whether transcription factors are involved in regulating these perturbed metabolic pathways, the differentially expressed transcription factors in the *Cg*Med2Δ strain relative to the wild-type strain were screened. Among these, *Cg*Yap6, *Cg*Hap5, *Cg*Com2, *Cg*Sut1, *Cg*Aft1, and *Cg*Yap5 were the most significantly differentially expressed transcription factors at pH 5.5 and pH 2.0 (Table 1 and Fig. 2C and D). Therefore, we further assessed the resistance of C. glabrata to low pH (2.0) upon deletion or overexpression of these transcription factors. Interestingly, deletion of *Cg*Yap6 and *Cg*Aft1 led to evident growth defects, whereas only overexpression of *Cg*Yap6 conferred resistance to acidic pH (Fig. 2E and F). Med2, a subunit of the tail module, interacts with transcription factors to regulate the transcription of nearly all RNA polymerase II-dependent genes in yeast (24). Hence, we hypothesized that the interaction between *Cg*Yap6 and *Cg*Med2 might play an important role in yeast resistance at pH 2.0.

**TABLE 1:** Differentially expressed genes associated with transcription factors and protein kinase

Gene name	S. cerevisiae homolog	Gene product function	Log_2_FC^*a*^ (*Cg*Med2Δ strain vs wt strain) at pH:	
			5.5	2.0
*CAGL0M08800g*	YAP6	Basic leucine zipper transcription factor; computational analysis suggests a role in regulation of expression of genes involved in carbohydrate metabolism	−0.93	−1.79
*CAGL0K09900g*	HAP5	Subunit of the Hap2p/3p/4p/5p CCAAT-binding complex; complex is a transcriptional activator and global regulator of respiratory gene expression	−0.96	−1.39
*CAGL0K02145g*	COM2	Transcription factor that binds IME1 upstream activation signal; C. albicans homolog (MNL1) plays a role in adaptation to stress	−0.89	−1.15
*CAGL0H03487g*	AFT1	Transcription factor involved in iron utilization and homeostasis	−0.32	−1.60
*CAGL0I04246g*	SUT1	Positively regulates sterol uptake genes under anaerobic conditions; involved in hypoxic gene expression	−0.77	−1.56
*CAGL0K08756g*	YAP5	Basic leucine zipper iron-sensing transcription factor; involved in diauxic shift	−0.62	−2.21
*CAGL0M13189g*	MSN2	Stress-responsive transcriptional activator; activated in stochastic pulses of nuclear localization in response to various stress conditions	−1.65	−1.51
*CAGL0F09075g*	SCH9	Protein kinase; involved in transactivation of osmostress-responsive genes; integrates nutrient signals and stress signals from sphingolipids to regulate lifespan	−0.58	−1.54
*CAGL0I05896g*	YAK1	Serine-threonine protein kinase; component of a glucose-sensing system that inhibits growth in response to glucose availability	−0.86	−2.46

a FC, fold change, which represents the ratio of the expression levels for two samples.

### *Cg*Med2 interacts with transcription factors at pH 2.0.

**FIG 3:**
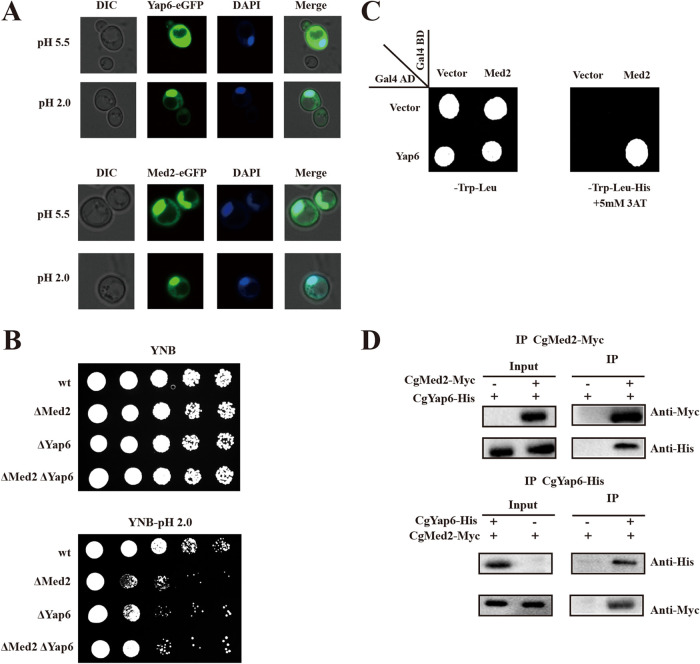
*Cg*Med2 interacts with *Cg*Yap6. (A) *Cg*Med2 and *Cg*Yap6 were fused with the eGFP reporter and overexpressed, and the subcellular localization was visualized under pH 2.0 and pH 5.5 conditions. (B) The wild-type, *Cg*Med2Δ, *Cg*Yap6Δ, and *Cg*Med2ΔYap6Δ strains were spotted on YNB plates under pH 2.0 and pH 5.5 conditions. (C) Yeast two-hybrid assays confirmed the interaction between *Cg*Med2 and *Cg*Yap6 at pH 2.0. (D) Coimmunoprecipitation assay to detect the interaction between *Cg*Yap6 and *Cg*Med2 *in vivo* at pH 2.0.

At pH 5.5, *Cg*Yap6 fused with enhanced green fluorescent protein (*Cg*Yap6-eGFP) localized in both the nucleus and cytoplasm, whereas at pH 2.0, it was detected mostly in the nucleus. In contrast, *Cg*Med2-eGFP was located in the nucleus at both pH 5.5 and pH 2.0 (Fig. 3A). These results indicated that the distribution of *Cg*Yap6-eGFP in the nucleus increases at pH 2.0, suggesting that *Cg*Med2 and *Cg*Yap6 may cooperate in the nucleus in response to acid pH stress.

To confirm this hypothesis, the genetic interaction between *Cg*Med2 and *Cg*Yap6 was evaluated using spot assays, which revealed that the phenotype of the *Cg*Med2Δ/*Cg*Yap6Δ double mutant was similar to those of the *Cg*Med2Δ and *Cg*Yap6Δ single mutants (Fig. 3B). Moreover, the *Cg*Med2Δ/*Cg*Yap6Δ double mutant showed 42.3% survival, whereas the *Cg*Med2Δ and *Cg*Yap6Δ single mutants exhibited 44.6% and 47.5% survival, respectively (Table 2). These results suggest a mechanism by which *Cg*Med2 and *Cg*Yap6 work together either in the same pathway or as part of the same protein complex to promote growth under a condition of low pH.

**TABLE 2:** Survival of various strains at pH 5.5 and pH 2.0

Strain	% survival^*a*^ at pH:	
	5.5	2.0
Wild type	100	77.4 (2.23)^*b*^
*Cg*Med2Δ	98.3 (1.34)	44.6 (3.41)^*b*^
*Cg*Yap6Δ	99.6 (1.62)	47.5 (2.57)^*b*^
*Cg*Med2Δ/*Cg*Yap6Δ	96.4 (1.47)	42.3 (1.95)^*b*^

a Survival rates are expressed relative to those of wild-type cells. Results are the averages from three experiments, with standard deviations in parentheses.

b *P* ≤ 0.01 versus wild type.

Next, the physical interaction between *Cg*Med2 and *Cg*Yap6 was determined at pH 5.5 (see Fig. S2 in the supplemental material) and pH 2.0 (Fig. 3C). Yeast two-hybrid (Y2H) analysis revealed a physical interaction between Gal4-BD (DNA-binding domain)-*Cg*Med2 and Gal4-AD (activation domain)-*Cg*Yap6 at pH 2.0 (Fig. 3C). This interaction was also confirmed by coimmunoprecipitation experiment (Fig. 3D; see Fig. S3 in the supplemental material), which showed that *Cg*Med2-Myc coprecipitated with *Cg*Yap6-His and vice versa at pH 2.0 (Fig. 3D).

These observations suggest that *Cg*Med2 interacts with CgYap6 at pH 2.0.

### *Cg*Yak1 is required for the pH-induced phosphorylation of *Cg*Yap6.

**FIG 4:**
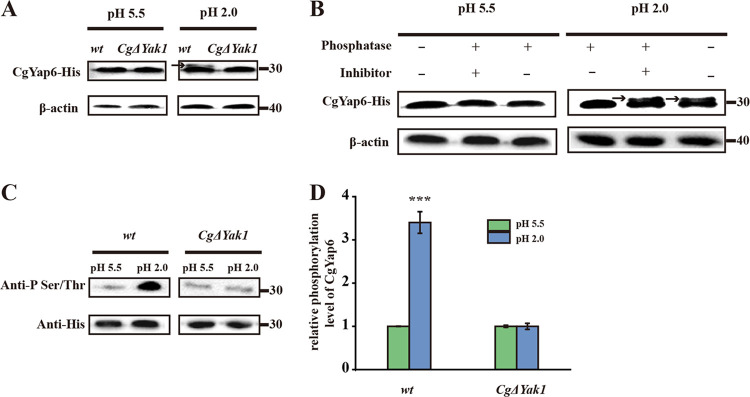
*Cg*Yak1 phosphorylates *Cg*Yap6 at pH 2.0. (A) Immunoprecipitation of *Cg*Yap6-His was performed in the wild-type (wt) and *Cg*Yak1Δ strains at pH 5.5 and pH 2.0, followed by Western blotting using anti-His antibody. The arrow indicates the phosphorylation band of *Cg*Yap6. (B) Extracts prepared from *Cg*Yap6-His-expressing wild-type cells, grown at pH 5.5 and pH 2.0, were treated with alkaline phosphatase and phosphatase inhibitor as indicated. The arrows indicate the phosphorylation band of *Cg*Yap6. (C) Immunoprecipitation of phosphorylated *Cg*Yap6 was performed in the wild-type (wt) and *Cg*Yak1Δ strains at pH 5.5 and pH 2.0, followed by Western blotting using anti-phosphoserine/threonine antibody. (D) Quantification of relative phosphorylation levels of *Cg*Yap6 in the wild-type (wt) and *Cg*Yak1Δ strains. Error bars indicate the standard deviations. *, *P* < 0.05; **, *P* < 0.01; ***, *P* < 0.001 (compared to the corresponding wild-type strain, as determined by a *t* test).

Based on the results presented above, we thought that transcription factors may undergo posttranslational modifications at pH 2.0 and then enter the nucleus to recruit the mediator *Cg*Med2 for transcriptional regulation. The protein kinase Yak1, which plays a central role in the regulation of many biological aspects in eukaryotic organisms (31), was differentially expressed in the *Cg*Med2Δ strain compared with the wild-type strain at pH 2.0 (Table 1). Therefore, we investigated whether *Cg*Yak1 phosphorylated *Cg*Yap6 at pH 2.0. According to gel electrophoresis results, *Cg*Yap6-His was expressed with the expected molecular mass of 31 kDa in the wild-type strain at pH 5.5; however, its weight shifted at pH 2.0 (Fig. 4A). Treatment with alkaline phosphatase showed that the band corresponding to *Cg*Yap6-His appeared at 31 kDa, whereas it shifted again when alkaline phosphatase inhibitor was added (Fig. 4B). These data support the hypothesis that the shift of the *Cg*Yap6-His band observed at pH 2.0 is the result of phosphorylation.

Additionally, for the *Cg*Yak1Δ strain, a *Cg*Yap6-His molecular weight change was not observed, indicating that the phosphorylation of *Cg*Yap6-His detected at pH 2.0 depended on the presence of *Cg*Yak1. To further verify these results, the phosphorylation of *Cg*Yap6 by *Cg*Yak1 was analyzed in both wild-type and *Cg*Yak1Δ strains by reaction with an anti-phosphoserine/threonine antibody, which is used to detect the phosphorylation of Ser-Pro/Thr-Pro (SP/TP) sites (Yak1 consensus phosphorylation sites). At pH 5.5, a weak *Cg*Yap6-His phosphorylation band was detected in both strains; however, at pH 2.0, the phosphorylation level of *Cg*Yap6-His increased obviously in the wild-type strain but not in the *Cg*Yak1Δ strain (Fig. 4C and D).

### Transcriptome and untargeted metabolomics analysis of the wild-type and *Cg*Med2Δ strains at pH 5.5 and pH 2.0.

**FIG 5:**
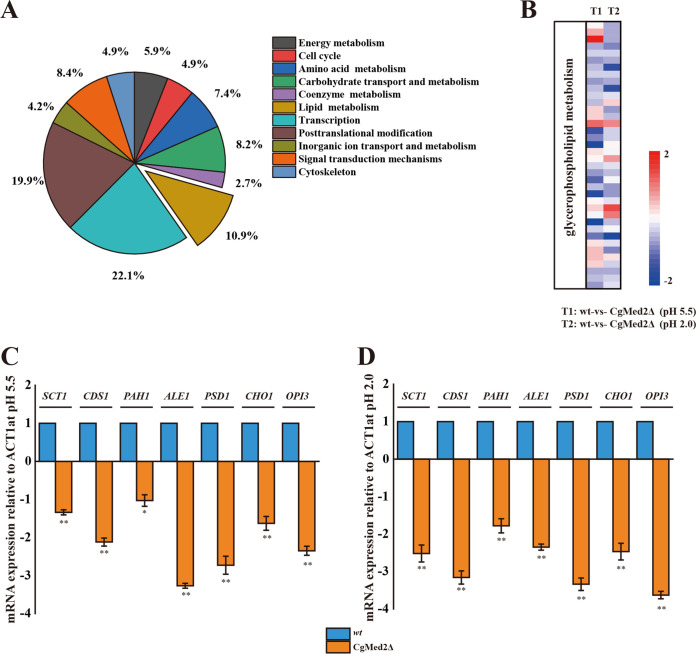
*Cg*Med2 is involved in regulating glycerophospholipid metabolism. (A) Statistical analysis of the metabolic pathways in which the differentially expressed genes were significantly enriched in the *Cg*Med2Δ strain compared with levels in the wild-type (wt) strain at pH 2.0. (B) Heat maps of differentially expressed genes involved in glycerophospholipid metabolism. (C and D) Quantitative reverse transcription-PCR (qRT-PCR) verified the mRNA expression levels of the glycerophospholipid genes, calculated relative to the *ACT1* level, under normal (C) and low-pH (D) conditions. Error bars indicate the standard deviations. *, *P* < 0.05; **, *P* < 0.01; ***, *P* < 0.001 (compared to the corresponding wild-type strain, as determined by a *t* test).

Functional enrichment analysis was performed on up- and downregulated differentially enriched KEGG pathways. Transcription, posttranslational modifications, lipid metabolism, signal transduction mechanisms, and carbohydrate metabolism were the five most highly enriched pathways (as mapped in the KEGG), accounting for 22.1%, 19.9%, 10.9%, 8.4%, and 8.2%, respectively, of all differentially expressed genes in the *Cg*Med2Δ strain compared to the wild-type strain at pH 2.0 (Fig. 5A). Specifically, lipid metabolism was the module exhibiting the largest variation of intracellular metabolic pathways. Consequently, the expression level of genes related to lipid metabolism was compared between the wild-type and *Cg*Med2Δ strains at pH 2.0 (Fig. 5B). The mRNA levels of genes involved in glycerophospholipid metabolism were analyzed by quantitative reverse transcription-PCR (qRT-PCR). At pH 5.5, the mRNA levels of glycerol-3-phosphate-*O*-acyltransferase (*SCT1*), phosphatidate cytidylyltransferase (*CSD1*), lysophospholipid acyltransferase (*ALE1*), CDP-diacylglycerol-serine-*O*-phosphatidyltransferase (*CHO1*), phosphatidylserine decarboxylase (*PSD1*), and phosphatidyl-*N*-dimethylethanolamine *N*-methyltransferase (*OPI3*) were 1.3-, 2.1-, 3.2-, 2.7-, 1.6-, and 2.3-fold lower in the *Cg*Med2Δ strain than in the wild-type strain, respectively, whereas the mRNA level of phosphatidate phosphatase (*PAH1*) remained unchanged (Fig. 5C). Conversely, at pH 2.0, the mRNA levels of those genes were 2.5-, 3.1-, 1.7-, 2.3-, 3.4-, 2.5-, and 3.6-fold lower in the *Cg*Med2Δ strain than in the wild-type strain (Fig. 5D). These data suggested that the expression of genes involved in glycerophospholipid biosynthesis strongly depends on the presence of *Cg*Med2.

**FIG 6:**
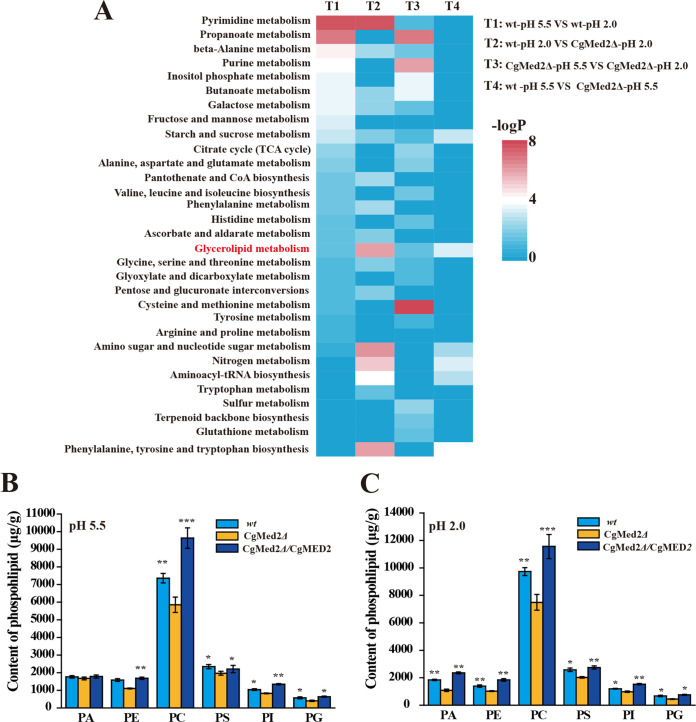
Untargeted metabolomics analysis of the wild-type strain and mutant *Cg*Med2Δ. (A) The results were combined from hydrophilic metabolomics extraction by LC-MS (including negative ionization and positive ionization). (B and C) Glycerophospholipid composition changes in the wild-type (wt), *Cg*Med2Δ, and *Cg*Med2Δ/*Cg*MED2 strains at pH 5.5 and pH 2.0. All data are presented as mean values from three independent experiments. Error bars indicate the standard deviations. *, *P* < 0.05; **, *P* < 0.01; ***, *P* < 0.001 (compared to the corresponding wild-type strain, as determined by a *t* test).

To explore the intracellular metabolism of the wild-type and *Cg*Med2Δ strains, we performed untargeted metabolomics for both the wild-type and *Cg*Med2Δ strains growing at pH 5.5 and pH 2.0. The results showed 24 and 20 differentially perturbed metabolic pathways in the wild‐type strain and *Cg*Med2Δ strain, respectively. Among those, 17 were common to both the wild-type and *Cg*Med2Δ strains. Those pathways were mostly related to amino acid metabolism, pyrimidine metabolism, amino sugar and nucleotide sugar metabolism, glycerolipid metabolism, and respiration (i.e., tricarboxylic acid [TCA] cycle and glyoxylate cycle). Moreover, untargeted metabolomics of wild-type and *Cg*Med2Δ strains under pH 2.0 stress were compared (see Table S1 in the supplemental material). At pH 5.5 and pH 2.0, 17 and 13 pathways, respectively, were perturbed in *Cg*Med2Δ compared with the wild-type strain. Among those perturbed pathways, 7 pathways were common in both the wild-type and *Cg*Med2Δ strains. These pathways were mostly related to amino acid metabolism, purine metabolism, glutathione metabolism, and glycerolipid metabolism. Although several of those perturbed pathways were related to amino acid metabolism and carbohydrate metabolism, metabolomics analysis identified glycerolipid metabolism as the only lipid pathway considerably perturbed in *Cg*Med2Δ under all conditions compared to the wild-type strain at pH 5.5 (Fig. 6A).

Based on the metabolomics results, the glycerophospholipid composition in the wild-type, *Cg*Med2Δ, and *Cg*Med2Δ/*Cg*MED2 strains was analyzed. At pH 5.5, the content of phosphatidic acid (PA), phosphatidylethanolamine (PE), phosphocholine (PC), phosphatidylserine (PS), phosphatidylinositol (PI), and phosphatidylglycerol (PG) decreased by 4.8%, 30.3%, 20.4%, 10.8%, 20.3%, and 29.6% in the *Cg*Med2Δ strain compared with those in the wild-type strain. In the *Cg*Med2Δ/*Cg*MED2 strain, the PE, PC, PS, PG, and PI content increased by 6.1%, 30.9%, 6.2%, 28.4%, and 11.2%, respectively, whereas the PA content remained unchanged (Fig. 6B). At pH 2.0, the content of PA, PE, PC, PS, PI, and PG decreased by 41.0%, 26.1%, 23.2%, 21.4%, 18.2%, and 33.8%, respectively, in the *Cg*Med2Δ strain compared with those in the wild-type strain. However, in the *Cg*Med2Δ/*Cg*MED2 strain, the PA, PE, PC, PS, PI, and PG content increased by 27.8%, 32.2%, 18.7%, 6.5%, 27.3%, and 12.6%, respectively, compared with those in wild-type strain (Fig. 6C). These results suggest that *Cg*Med2 may be critical for regulating glycerophospholipid composition at pH 2.0.

### *Cg*Yap6 regulates glycerophospholipid genes in a *Cg*Yak1-dependent manner.

**FIG 7:**
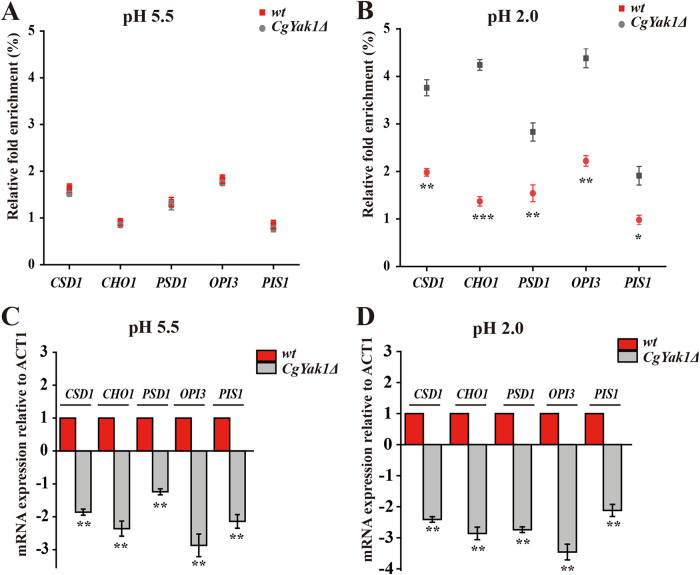
*Cg*Yap6 regulates glycerophospholipid genes in a *Cg*Yak1-dependent manner. (A and B) Association of *Cg*Yap6 with the core promoter of glycerophospholipid metabolism genes was determined by ChIP analysis combined with qRT-PCR to measure occupancy in the wild-type (wt) and *Cg*Yak1Δ strains at pH 5.5 and pH 2.0. Relative fold enrichment was calculated by the formula provided in Materials and Methods. (C and D) Transcript levels of genes involved in glycerophospholipid metabolism were analyzed with RNA prepared from the wt and *Cg*Yak1Δ strains at pH 5.5 and pH 2.0. The data were normalized to the expression level of the *ACT1* gene. All data are presented as mean values from three independent experiments. Error bars indicate the standard deviations. *, *P* < 0.05; **, *P* < 0.01; ***, *P* < 0.001 (compared to the corresponding wild-type strain, as determined by a *t* test).

To reveal the regulatory circuitry among *Cg*Med2, *Cg*Yak1, and *Cg*Yap6, we assessed whether the phosphorylation of *Cg*Yap6 is required for its function in regulating genes involved in glycerophospholipid metabolism. A chromatin immunoprecipitation (ChIP) assay combined with qRT-PCR was performed to detect the binding of *Cg*Yap6 to glycerophospholipid genes in the wild-type and *Cg*Yak1Δ strains. The qRT-PCR analysis revealed that at pH 5.5, the levels of binding of *Cg*Yap6 to the promoter regions of *CSD1*, *CHO1*, *PSD1*, *OPI3*, and *PIS1* were not significantly different between the wild-type and *Cg*Yak1Δ strains (Fig. 7A), whereas the binding levels were, respectively, 47.3%, 67.6%, 45.5%, 49.3%, and 48.6% lower in the *Cg*Yak1Δ strain than in the wild-type strain at pH 2.0 (Fig. 7B). These data demonstrate that the *Cg*Yak1 target transcription factor *Cg*Yap6 promotes the binding of *Cg*Yap6 to target promoters at pH 2.0. Moreover, the transcription levels of *CSD1*, *CHO1*, *PSD1*, *OPI3*, and *PIS1* were 1.8-, 2.3-, 1.2-, 2.9-, and 2.1-fold lower in the *Cg*Yak1Δ strain than in the wild-type strain at pH 5.5 (Fig. 7C) and were 2.4-, 2.8-, 2.7-, 3.4-, and 2.0-fold lower in the *Cg*Yak1Δ strain than in the wild-type strain at pH 2.0, respectively (Fig. 7D). These results suggest that the *Cg*Yak1 target transcription factor *Cg*Yap6 is indeed important for enhancing the transcription levels of glycerophospholipid genes.

### *Cg*Med2 affects membrane integrity at pH 2.0.

**FIG 8:**
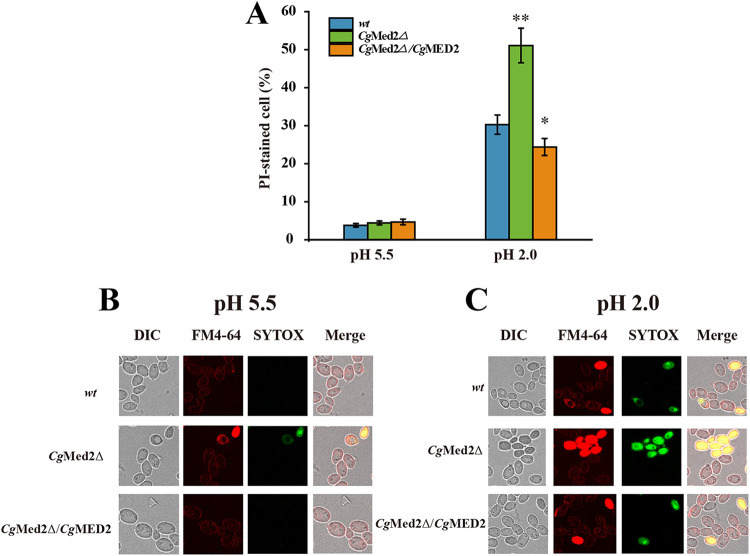
*Cg*Med2 affects membrane integrity. (A) Quantification of membrane integrity in the wild-type, *Cg*Med2Δ, and *Cg*Med2Δ/*Cg*MED2 strains at pH 5.5 and pH 2.0. (B and C) Scanning electron microscopy analysis of membrane integrity in the wild-type, *Cg*Med2Δ, and *Cg*Med2Δ/*Cg*MED2 strains at pH 5.5 and pH 2.0. Under a confocal fluorescence microscope, all cells showed red fluorescence indicating an integral membrane, whereas cells with a damaged membrane showed green fluorescence (SYTOX green); cells with integral or damaged membranes can be stained by FM4-64. Error bars indicate the standard deviations. *, *P* < 0.05; **, *P* < 0.01; ***, *P* < 0.001 (compared to the corresponding wild-type strain, as determined by a *t* test).

To describe the role of *Cg*Med2 in membrane integrity, cells of the wild-type, *Cg*Med2Δ, and *Cg*Med2Δ/*Cg*MED2 strains were cultivated at both pH 5.5 and 2.0 for 3 h, followed by propidium iodide uptake analysis. At pH 5.5, approximately 3.8%, 4.4%, and 4.7% of the wild-type, *Cg*Med2Δ, and *Cg*Med2Δ/*Cg*MED2 cells were stained, respectively. At pH 2.0, the proportion of stained cells increased to 20.8% in the *Cg*Med2Δ strain but decreased by 5.9% in the *Cg*Med2Δ/*Cg*Med2 strain compared with those in the wild-type strain (Fig. 8A; see Fig. S8 in the supplemental material). These results suggest that *Cg*Med2 contributes to increasing membrane integrity at pH 2.0.

To visually observe membrane integrity, the wild-type, *Cg*Med2Δ, and *Cg*Med2Δ/*Cg*MED2 strains were cultivated at both pH 5.5 and 2.0 for 3 h, followed by SYTOX green and FM4-64 uptake analysis observed with confocal laser scanning microscopy observations. At pH 5.5, almost all cells of the three strains had integral membranes (Fig. 8B). At pH 2.0, some cells of the wild-type, *Cg*Med2Δ, and *Cg*Med2Δ/*Cg*MED2 strains displayed damaged membranes, although the damage was the least apparent for the *Cg*Med2Δ/*Cg*MED2 strain, followed by the wild-type strain, and the *Cg*Med2Δ strain showed the highest number of cells with damaged membranes (Fig. 8C). These results suggested that *Cg*Med2 plays an important role in membrane integrity.

## DISCUSSION

In this study, we investigated the role of *Cg*Med2 in the response to pH 2.0 stress. Our results suggest that this stress response prompts *Cg*Yak1 to target the transcription factor *Cg*Yap6, followed by its translocation from the cytoplasm to the nucleus. Inside the nucleus, phosphorylated *Cg*Yap6 recruits *Cg*Med2 to induce the expression of genes involved in the glycerophospholipid pathway, resulting in membrane integrity enhancement.

Previous studies have indicated that Med2 regulates cell growth under stress conditions. In Candida albicans, Med2 affects the ability to form adherent biofilms and invasive hyphae to resist various environmental and nutrient stresses (30, 32). In Candida dubliniensis, Tlo1 (a Med2 ortholog) is required for the response to carbon source and oxidative stress (33). In this study, C. glabrata lacking Med2 exhibited a severe growth defect at pH 2.0, whereas overexpression of *Cg*Med2 enhanced cell growth and survival rates compared to those of the wild-type strain. These results suggest that Med2 may play different roles under various conditions of environmental stress. Furthermore, a previous study on C. glabrata reported that *Cg*Med3 plays an important role in the response to acid pH stress, by regulating the expression of genes involved in lipid metabolism, ribosome biosynthesis, and carbohydrate metabolism (20). Our transcriptome analysis demonstrated that in the *Cg*Med2 deletion strain, genes involved in the translation process, posttranslational modification, lipid metabolism, and carbohydrate metabolism were downregulated at pH 2.0, suggesting that *Cg*Med2 and *Cg*Med3 function redundantly at acid pH but that deletion of individual tail subunits causes different effects on gene expression.

The protein kinase Yak1 regulates a variety of cellular functions, such as cell growth and stress response. Previous studies reported that Yak1 is involved in resistance to oxidative stress (34) and organic acid stress (35). In addition, inactivation of Yak1 causes abnormal polarized apical growth in Trichoderma reesei, as well as sensitivity to various stresses (36). In this report, we highlighted a new physiological function of Yak1 in C. glabrata. Specifically, *Cg*Yak1 phosphorylates the transcription factor Yap6 in response to acid pH stress. In S. cerevisiae, Yak1 substrates include transcription factors, metabolic enzymes, and other protein kinases (31). Therefore, transcription factors can be phosphorylated by Yak1 under stress conditions (37, 38). For example, in S. cerevisiae, the conserved protein kinase Yak1 was activated under heat stress, and its target transcription factors (i.e., Msn2 and Msn4) were phosphorylated, thereby affecting DNA-binding activity. Perturbation of this pathway considerably affected cell survival upon heat stress (38). These findings are consistent with our results that *Cg*Yak1 targeted *Cg*Yap6 to induce its translocation from the cytoplasm to the nucleus under pH 2.0 stress. It has been reported that nutrient starvation acts as a cellular signal to also activate Yak1 (39). This led us to speculate that the absorption of nutrients by C. glabrata is hampered under pH 2.0 stress, and that *Cg*Yak1 then targets the transcription factor *Cg*Yap6 to promote its translocation to the cell nucleus, where it performs its functions.

Here, we demonstrated that *Cg*Yap6 can recruit *Cg*Med2 to regulate the expression of glycerophospholipid genes at pH 2.0. The subunits of the tail module regulate the expression of genes involved in diverse metabolic pathways by either directly or indirectly interacting with their respective transcriptional activators (25). For example, the Med15 subunit of the tail module has been shown to regulate the expression of genes involved in fatty acid metabolism by directly interacting with the activator Oaf1 in S. cerevisiae (40). Similar results have also been obtained in human cells and Caenorhabditis elegans (41, 42). This study provides a new link between the tail module of the *Cg*Med2 subunit and the transcription factor *Cg*Yap6 at pH 2.0. Our results confirm that the tail module functions mainly as a target for transcription factors to active gene expression. Several studies have proposed that some transcription factors can recruit Med2 to resist environmental stress (43, 44). In C. glabrata, the increased susceptibility of *Cg*Med2 mutants to azole antifungal drugs is due to the inability to activate the zinc finger transcription factor *Cg*Pdr1 to transcribe multidrug efflux pumps *Cg*Cdr1 (43). In addition, several pieces of evidence suggest that some transcription factors require posttranslational modifications to be activated in order to recruit Med2 (45, 46). For example, under arsenate stress, the cysteine sulfhydryl group of Yap8 is modified into its activated form. This active form interacts with the tail subunit Med2, which facilitates the Acr2/Acr3 promoter recruitment of the Mediator complex, leading to upregulation of Acr2 and cell tolerance to the stressor (45). This previous result recalls our data, according to which *Cg*Yak1-mediated phosphorylation of *Cg*Yap6 facilitates the recruitment of the mediator *Cg*Med2 to regulate downstream glycerophospholipid gene expression and glycerophospholipid composition in response to pH 2.0 stress (see Fig. S5 in the supplemental material). In industrial microorganisms, adverse environmental pressure often causes damage to the cell membrane (47, 48). Enhancement of the phospholipid content has been proven to be effective in increasing cell membrane integrity and stress tolerance. For example, in Escherichia coli, overexpression of phosphatidylserine synthetase (PssA) increased the content of PE in the cell membrane, thereby enhancing the bacterium’s tolerance to octanoic acid and increasing yield (49). In S. cerevisiae, modularized engineering was used to change the composition of the phospholipid head, thereby increasing the integrity of the cell membrane and improving salt tolerance (50). These results indicate that alteration of the phospholipid content in the cell membrane is a potential strategy to improve the robustness of industrial microorganisms.

In conclusion, when C. glabrata is subjected to acid pH stress, the protein kinase *Cg*Yak1 integrates the stress signals and transmits them to the transcription factor *Cg*Yap6. Specifically, *Cg*Yak1 induces the translocation of *Cg*Yap6 from the cytoplasm to the nucleus, where it binds to the promoter region of glycerophospholipid-related genes and then recruits *Cg*Med2 to regulate the glycerophospholipid composition of the cell membrane and its integrity. Therefore, this study may provide a potential strategy for enhancing the resistance of C. glabrata to acid pH stress during organic acid fermentation.

## MATERIALS AND METHODS

### Strains, media, and culture conditions.

The yeast strains and plasmids used in this study are listed in Table 3. Escherichia coli JM109 was used for cloning and plasmid propagation. All yeast strains used in this study were derived from a C. glabrata *Cg*HTUΔ (*his3*Δ *trp1*Δ *ura3*Δ) strain. The deletion strains were constructed by homologous recombination of *Cg*HIS3, *Cg*TRP1, and *Cg*URA3 markers in the *Cg*Med2, *Cg*Yap6, and *Cg*Yak1 loci. The marker genes were amplified from the C. glabrata strain ATCC 2001 genome and fused between the upstream and downstream regions of the *Cg*Med2, *Cg*Yap6, and *Cg*Tpk1 gene open reading frames by fusion PCR. PCR products were transformed in the C. glabrata strain *Cg*HTUΔ as described previously, and the deletion strains were confirmed by genomic PCR and DNA sequencing.

**TABLE 3:** Strains and plasmids used in this study

Strain or plasmid	Relevant characteristics	Reference
Strains		
C. glabrata ATCC 2001		51
C. glabrata HTUΔ	*his3*Δ *trp1*Δ *ura3*Δ	51
*Cg*Med2Δ	*his3*Δ *trp1*Δ *ura3*Δ *CgMED2*::*CgHIS3*	This study
*Cg*Med2Δ/*Cg*MED2	*his3*Δ *trp1*Δ *ura3*Δ *med2*Δ *CgMED2*::*CgHIS3* pY26-PGPD*/CgMED2*	This study
*Cg*Yap6Δ	*his3*Δ *trp1*Δ *ura3*Δ *CgYAP6*::*CgTRP1*	This study
*Cg*Hap5Δ	*his3*Δ *trp1*Δ *ura3*Δ *CgHAP5*::*CgHIS3*	This study
*Cg*Com2Δ	*his3*Δ *trp1*Δ *ura3*Δ *CgCOM2*::*CgHIS3*	This study
*Cg*Aft1Δ	*his3*Δ *trp1*Δ *ura3*Δ *CgAFT1*::*CgHIS3*	This study
*Cg*Sut1Δ	*his3*Δ *trp1*Δ *ura3*Δ *CgSUT1*::*CgHIS3*	This study
*Cg*Yap5Δ	*his3*Δ *trp1*Δ *ura3*Δ *CgYAP5*::*CgHIS3*	This study
*Cg*Yak1Δ	*his3*Δ *trp1*Δ *ura3*Δ *CgYAK1*::*CgHIS3*	This study
*Cg*Med2Δ*Cgyap6*Δ	*his3*Δ *trp1*Δ *ura3*Δ *CgYAP6*::*CgTRP1 CgMED2*::*CgHIS3*	This study
*Cg*Yap6Δ/*Cg*YAP6	*his3*Δ *trp1*Δ *ura3*Δ *yap6*Δ *CgYAP6*::*CgTRP1* pY26-PGPD*/CgYAP6*	This study
*Cg*Hap5Δ/*Cg*HAP5	*his3*Δ *trp1*Δ *ura3*Δ *hap5*Δ *CgHAP5*::*CgHIS3* pY26-PGPD*/CgHAP5*	This study
*Cg*Com2Δ/*Cg*COM2	*his3*Δ *trp1*Δ *ura3*Δ *com2*Δ *CgCOM2*::*CgHIS3* pY26-PGPD*/CgCOM2*	This study
*Cg*Aft1Δ/*Cg*AFT1	*his3*Δ *trp1*Δ *ura3*Δ *aft1*Δ *CgAFT1*::*CgHIS3* pY26-PGPD*/CgAFT1*	This study
*Cg*Sut1Δ/*Cg*SUT1	*his3*Δ *trp1*Δ *ura3*Δ *sut1*Δ *CgSUT1*::*CgHIS3* pY26-PGPD*/CgSUT1*	This study
*Cg*Yap5Δ/*Cg*YAP5	*his3*Δ *trp1*Δ *ura3*Δ *yap5*Δ *CgYAP5*::*CgHIS3* pY26-PGPD*/CgYAP5*	This study
AH109	*trp1*Δ *leu2*Δ *ura3*Δ *his3*Δ *gal4*Δ *gal80*Δ *LYS2*::*GAL1UAS-GAL1TATA-HIS3GAL2UAS-GAL2TATA-ADE2 URA3*::*MEL1UAS-MEL1TATA-LacZMEL1*	This study
*Cg*Med2Δ/*Cg*MED2-Myc	*his3*Δ *trp1*Δ *ura3*Δ *CgMED2*::*CgHIS3* pY26-PGPD*/Cg*Med2-Myc	This study
*Cg*Med2Δ/*Cg*Yap6Δ/*Cg*Med2-Myc/*Cg*Yap6-His	*his3*Δ *trp1*Δ *ura3*Δ *CgYAP6*::*CgTRP1 CgMED2*::*CgHIS3* pY26-PGPD*/Cg*Med2-Myc pY26-PTEF*/Cg*Yap6-His	This study
Plasmids		
pY26	2μ Amp *URA3* P_GPD_ P_TEF_	This study
pGBKT7	Kan *TRP1* GAL4 DNA-binding domain fusion	This study
pGADT7	Amp *LEU2* GAL4 DNA-binding domain fusion	This study

E. coli JM109 cells were grown in LB medium (2% tryptone, 2% NaCl, 1% yeast extract) and incubated at 37°C with shaking at 200 rpm. Ampicillin (100 mg/liter) was added for the selection of cells carrying the relevant plasmid. Unless otherwise stated, yeast strains were grown in yeast nitrogen base (YNB) (0.67% yeast nitrogen base, 2% glucose) medium supplemented with essential nutrients and incubated at 30°C with shaking at 200 rpm.

### Plasmids.

The *Cg*Med2 gene was amplified from C. glabrata genomic DNA using the primers listed in Table 4. For establishment of the overexpression strains, we used the *GPD* promoter of plasmid pY26 (copy number of 2μ plasmid); the target genes were amplified from the genome of C. glabrata using primers containing BamHI and HindIII restriction sites and cloned into pY26 to generate *Cg*Med2/*Cg*MED2, *Cg*Yap6/*Cg*YAP6, *Cg*Yak1/*Cg*YAK1, *Cg*Hap5/*Cg*HAP5, *Cg*Com2/*Cg*COM2, *Cg*Sut1/*Cg*SUT1, *Cg*Aft1/*Cg*AFT1, and *Cg*Yap5/*Cg*YAP5 strains. Clones were confirmed by DNA sequencing.

**TABLE 4:** Primers used in this study

Category and name	Sequence (5′→3′)^*a*^
Deletion	
L-*Cg*MED2-F1	TACAAATTAGCTATTATTACCAA
L-*Cg*MED2-F2	CTTAACAAACGCCATTACTTTCAGACGGGCAGTTTAT
*Cg*HIS3(*CgMed2*)-F1	GCCCGTCTGAAAGTAATGGCGTTTGTTAAGAGGGT
*Cg*HIS3(*CgMed2*)-F2	AATAGGAAGACCAAGCTATGCTAGGACACCCTTAG
R-*Cg*MED2-F1	GGTGTCCTAGCATAGCTTGGTCTTCCTATTGTTAACTATT
R-*Cg*MED2-F2	GGATAAATTTTTTGATAGTTTAGTA
L-*Cg*YAP6-F1	TTTATCAGGAACAAGCTGTTAC
L-*Cg*YAP6-F2	AGTAACGAATCAAATGACATTATATCTCCGGAAATATAGT
*Cg*TRP1(*CgYAP6*)-F1	ACTATATTTCCGGAGATATAATGTCATTTGATTCGTTACT
*Cg*TRP1(*CgYAP6*)-F2	AGTGCATTCGGAGTTCAGAGTCATTGTTTCTTTGCATTTTGTACA
R-*Cg*YAP6-F1	AAAATGCAAAGAAACAATGACTCTGAACTCCGAATGCACT
R-*Cg*YAP6-F2	AAAAGCATCTGTCACAGAAAAG
L-*Cg*HAP5-F1	TTTGTAGTGCCGCTTTCCC
L-*Cg*YAP5-F2	ACCCTCTTAACAAACGCCATTACCCACTTCTAATATATAAACCTG
*Cg*HIS3(*CgHap5*)-F1	TTATATATTAGAAGTGGGTAATGGCGTTTGTTAAGAGGGT
*Cg*HIS3(*CgHap5*)-F2	TCAGGATTACATACTACATACTATGCTAGGACACCCTTAG
R-*Cg*HAP5-F1	CTAAGGGTGTCCTAGCATAGTATGTAGTATGTAATCCTGAATAAG
R-*Cg*HAP5-F2	ACAACTTTTTCTTGGTATAATTTAT
L-*Cg*COM2-F1	CTTTCCTTTAATGCTCTCGA
L-*Cg*COM2-F2	ACCCTCTTAACAAACGCCATTACTCCGTAGTGTATAATTGT
*Cg*HIS3(*CgCom2*)-F1	CAATTATACACTACGGAGTAATGGCGTTTGTTAAGAGGGT
*Cg*HIS3(*CgCom2*)-F2	TTGCATTCAAGCAAATACCACTATGCTAGGACACCCTTAG
R-*Cg*COM2-F1	CTAAGGGTGTCCTAGCATAGTGGTATTTGCTTGAATGCAAAA
R-*Cg*COM2-F2	CCTCATTAAGTGATGACGAACTACA
L-*Cg*AFT1-F1	TATAAGGAAGGAAAAGTTAAGACGT
L-*Cg*AFT1-F2	ACCCTCTTAACAAACGCCATAGCATTTCAATATTTCAATAAGAAA
*Cg*HIS3(*CgAft1*)-F1	TATTGAAATATTGAAATGCTATGGCGTTTGTTAAGAGGGT
*Cg*HIS3(*CgAft1*)-F2	CATCTCATATCAATCAATATCTATGCTAGGACACCCTTAG
R-*Cg*AFT1-F1	CTAAGGGTGTCCTAGCATAGATATTGATTGATATGAGATGTATTG
R-*Cg*AFT1-F2	TCTGTACCATCTTTTATATGCAATC
L-*Cg*SUT1-F1	AAAAAGAAAAAGCTATAGCAAGGAG
L-*Cg*SUT1-F2	ACCCTCTTAACAAACGCCATTTCGTTAATTTTTAGTTTATGCTTT
*Cg*HIS3(*CgSut1*)-F1	ATAAACTAAAAATTAACGAAATGGCGTTTGTTAAGAGGGT
*Cg*HIS3(*CgSut1*)-F2	CTTTAGTTATCGATGGTAAACTATGCTAGGACACCCTTAG
R-*Cg*SUT1-F1	CTAAGGGTGTCCTAGCATAGTTTACCATCGATAACTAAAGCCTTA
R-*Cg*SUT1-F2	GCCTTTTGTGTTTAAGTTGC
L-*Cg*YAP5-F1	ATGCGAACCCTTCGCC
L-*Cg*YAP5-F2	ACCCTCTTAACAAACGCCATCGGGTATCCACCGCTAG
*Cg*HIS3(*CgYap5*)-F1	GGCCTAGCGGTGGATACCCGATGGCGTTTGTTAAGAGGGT
*Cg*HIS3(*CgYap5*)-F2	AAGCTATTCTACAGCGATAACTATGCTAGGACACCCTTAG
R-*Cg*YAP5-F1	CTAAGGGTGTCCTAGCATAGTTATCGCTGTAGAATAGCTTAATAT
R-*Cg*YAP5-F2	AAAACTGTGACTTTCTGCCT
L-*Cg*YAK1-F1	ACTATATCAGCAGCTACGAGCC
L-*Cg*YAK1-F2	CCCTCTTAACAAACGCCATCGAAGATAATCTGCCCCTTCA
*Cg*HIS3(*CgYak1*)-F1	GAAGGGGCAGATTATCTTCGATGGCGTTTGTTAAGAGGGT
*Cg*HIS3(*CgYak1*)-F2	TTAATGAAATGGAGATAAAACTATGCTAGGACACCCTTAG
R-*Cg*YAK1-F1	CTAAGGGTGTCCTAGCATAGTTTTATCTCCATTTCATTAAATCTT
R-*Cg*YAK1-F2	TGGGGACAACTCACTGGAT
Overexpression	
*CgMED2*-F1(pY26)	GATTCTAGAACTAGTGGATCCATGAGTTACAAGAACAGGCTTACGG
*CgMED2*-F2(pY26)	GTCGACGGTATCGATAAGCTTTTAGATATTAAAGCCATTTAGGTCTAGGTC
*CgYAP6*-F1(pY26)	GATTCTAGAACTAGTGGATCCATGGGACAAGTTAACATGCGACC
*CgYAP6*-F2(pY26)	GTCGACGGTATCGATAAGCTTCTAGGACTTCTCGCCAGCAATT
*CgHAP5*-F1(pY26)	GATTCTAGAACTAGTGGATCCATGGAGAAGATGGAAAAGACGTATG
*CgHAP5*-F2(pY26)	GTCGACGGTATCGATAAGCTTTTACGAAGAGTTGTTTTGCGCTC
*CgCOM2*-F1(pY26)	GATTCTAGAACTAGTGGATCCATGACGGACACATTTCAGCTGG
*CgCOM2*-F2(pY26)	GTCGACGGTATCGATAAGCTTTCAATGGCTATGTGTTTTTATGTGTT
*CgAFT1*-F1(pY26)	GATTCTAGAACTAGTGGATCCATGGATTCCAACCAACTAATACACTT
*CgAFT1*-F2(pY26)	GTCGACGGTATCGATAAGCTTTCACATTATGTGATCTTCTTCTAATTTAACA
*CgSUT1*-F1(pY26)	GATTCTAGAACTAGTGGATCCATGGCTACAAGTATAACTGTTTTGAATAGA
*CgSUT1*-F2(pY26)	GTCGACGGTATCGATAAGCTTTTAGAATCCTGCCTTCTTGTATTCC
*CgYAP5*-F1(pY26)	GATTCTAGAACTAGTGGATCCATGCTGACTGCTCTGGGATCA
*CgYAP5*-F2(pY26)	GTCGACGGTATCGATAAGCTTCTATGTTCTTTGTCTTTTCGGGG
Yeast two-hybrid assay	
BD-*CgMED2*-F1	TTGACTGTATCGCCGGAATTCATGAGTTACAAGAACAGGCTTACGG
BD-*CgMED2*-F2	CTATAGGGCTCTAGAGTCGACTTAGATATTAAAGCCATTTAGGTCTAGGTC
AD-*CgYAP6*-F1	AAAGAGATCGAATTAGGATCCATGGGACAAGTTAACATGCG
AD-*CgYAP6*-F2	AAGCATTAGAGAATTGAATTCCTAGGACTTCTCGCCAGCA
Coimmunoprecipitation	
pY26/PGPD-*CgMED2*-F1	ATTCTAGAACTAGTGGATCCATGAGTTACAAGAACAGGCT
pY26/PGPD-*CgMED2*-F1	TCGACGGTATCGATAAGCTTTTACAGATCCTCTTCAGAGATGAGTTTCTGCTCGATATTAAAGCCATTTAGGTCTAGG
pY26/PTEF-*CgYAP6*-F1	GAATTGTTAATTAAAGATCTCTAGTGGTGGTGGTGGTGGTGGGACTTCTCGCCAGCAATTG
pY26/PTEF-*CgYAP6*-F2	CAGTTAACTCCGGACCGCGGATGGGACAAGTTAACATGCG
RT-PCR	
*YAP5*-F1	GGAGGATTCCAAATGCTA
*YAP5*-F2	GTTGCTCAAGAATTCGTC
*HAP5*-F1	ACGAGATAGAGTCTACGA
*HAP5*-F2	GCGAATATTATAGGTGCC
*COM2*-F1	GAGGAACTACTTCAATAACATA
*COM2*-F2	CGTCATTAAGAGTCATTACA
*YAP6*-F1	GCATCAGTACACCACTAG
*YAP6*-F2	GACCATTTTCCGAAGAGA
*AFT1*-F1	GCCAGATCGACTAATAAC
*AFT1*-F2	TGGTGACATGTATATTGAC
*SUT1*-F1	CGTTGAGTAGTGTACCAA
*SUT1*-F2	AGCCTTCTTCAGTTCTAG
*SCT1*-F1	AGTGCATTGGTATTTTCC
*SCT1*-F2	TGAGGATGGAAGTAATTCA
*CDS1*-F1	ACCTGACTTGTGATTTGA
*CDS1*-F2	TTGGCTTAACGGTAATGA
*PAH1*-F1	CTCTGATGCTATTGATAAAGG
*PAH1*-F2	CACAGTACGATAGGACAG
*ALE1*-F1	GCTGAAGAGATTGCCTAA
*ALE1*-F2	AGTCGCATAGGTGAATAG
*PSD1*-F1	CCGAAGAGACTAACCTATA
*PSD1*-F2	CCAGTTTATTGGTGAATGA
*CHO1*-F1	AAGGGCAAGTCTAAGTTC
*CHO1*-F2	GCAAGAAGAAGATGAAGATG
*OPI3*-F1	GCTCCATACTTCTACTCTG
*OPI3*-F2	CAGCAATCTTGGTTAGGA

a Underlining indicates sequences of regions flanking a target gene or a restriction site.

The *Cg*Yap6-His and *Cg*Med2-Myc constructs were amplified from C. glabrata genomic DNA using the primers listed in Table 4. *Cg*Med2-Myc was cloned into the vector pY26 using the NotI and BglII restriction sites, and the construct was named pY26-*Cg*Med2-Myc. CgYap6-His was cloned into the plasmid pY26-*Cg*Yap6-Myc using the HindIII restriction site and a ClonExpress II one-step cloning kit (C112-01; Vazyme), and the construct was named pY26-*Cg*Med2-Myc-*Cg*Yap6-His. Similarly, clones were confirmed by DNA sequencing.

The *Cg*Yap6 and *Cg*Med2 genes were amplified from C. glabrata genomic DNA using the primers listed in Table 4. The *Cg*Med2 gene was cloned into the vector pGBKT7 using the ClonExpress II one-step cloning kit (C112-01; Vazyme), and the construct was named pGBKT7-*Cg*Med2. The *Cg*Yap6 gene was cloned into the vector pGADT7 using the ClonExpress II one-step cloning kit (C112-01; Vazyme), and the construct was named pGADT7-*Cg*Yap6.

### Spot assay.

Yeast cells were cultivated in logarithmic phase and diluted to an absorbance at 660 nm (*A*_660_) of 1.0 in phosphate-buffered saline (PBS). Aliquots of 10-fold serial dilutions were spotted onto YNB agar plates with the indicated concentration of HCl. Growth was assessed after incubation for 2 to 4 days at 30°C.

### Growth and survival analysis.

To analyze the growth of C. glabrata strains at pH 2.0, logarithmic-phase cells were diluted into fresh YNB medium at pH 5.5 or 2.0 at an initial *A*_660_ of 0.1. Cultures were taken at regular time intervals, and the *A*_660_ values were recorded. The *A*_660_ was calibrated against the dry weight of cells (DCW) on the basis of a standard curve where an *A*_660_ of 1 is equal to a DCW of 0.23 g/liter. To analyze cell survival, yeast cells were cultivated in logarithmic phase and then treated with various concentrations of HCl for 1 h at 30°C with shaking at 200 rpm. Cells were then centrifuged and washed with sterile water three times. After dilution, cells were plated on YNB medium plates with the same number from each concentration of HCl and incubated at 30°C for 2 to 4 days. The surviving colonies were then counted. Data are presented as a percentage relative to untreated cells of the corresponding strain. The half-maximal inhibitory concentration (IC_50_) was calculated by fitting a Hill-type model to the data.

### RNA extraction.

C. glabrata cells were cultured to logarithmic phase and then reinoculated into fresh YNB medium at an initial *A*_660_ of 0.1. After incubation for 6 h, cells were harvested and released to YNB medium at pH 5.5 and pH 2.0 for 2 h. The experiments were performed in biological triplicate. Cells were then recollected and washed twice with phosphate-buffered saline by resuspension and centrifugation at 3,500 × *g* for 10 min at 4°C. Total RNA was isolated by using a MiniBEST universal RNA extraction kit (TaKaRa Bio, Shiga, Japan). The concentration and quality of total RNA were determined by microspectrophotometry using an Agilent 2100 Bioanalyzer (Agilent Technologies, Santa Clara, CA).

### RNA-seq analysis.

A transcriptome sequencing (RNA-seq) transcriptome library was prepared with a TruSeq RNA sample preparation kit from Illumina (San Diego, CA) using 1 μg of total RNA. Briefly, mRNA was isolated according to the poly(A) selection method with oligo(dT) beads and then fragmented by fragmentation buffer. Next, double-stranded cDNA was synthesized using a SuperScript double-stranded cDNA synthesis kit (Invitrogen, CA) with random hexamer primers (Illumina). The synthesized cDNA the was subjected to end repair, phosphorylation, and “A” base addition according to Illumina’s library construction protocol. Libraries were size selected for cDNA target fragments of 300 bp on 2% Low Range Ultra agarose, followed by PCR amplification using Phusion DNA polymerase (NEB) for 15 PCR cycles. After quantified by TBS380, the paired-end RNA-seq library was sequenced with the Illumina HiSeq xten/NovaSeq 6000 sequencer (2 × 150-bp read length). The raw paired-end reads were trimmed and quality controlled by SeqPrep (https://github.com/jstjohn/SeqPrep) and Sickle (https://github.com/najoshi/sickle) with default parameters. Clean reads (see Table S2 in the supplemental material) then were aligned to the reference genome of Candida glabrata CBS 138 (https://www.ncbi.nlm.nih.gov/genome/192?genome_assembly_id=28426). The differential expression analysis was performed using the DESeq software.

### qRT-PCR analysis.

C. glabrata cells were cultured as described for transcriptome sequencing analysis. Total RNA was extracted using a MiniBEST universal RNA extraction kit (TaKaRa Bio, Shiga, Japan), and 1 μg was used to synthesize cDNA with a PrimeScript II first-strand cDNA synthesis kit (TaKaRa Bio, Shiga, Japan). The cDNA mixture was diluted to approximately 100 ng/μl and used for quantitative real-time PCR (qRT-PCR) with TB Green Premix *Ex Taq* (TaKaRa Bio, Shiga, Japan) on an iQ5 continuous fluorescence detector system (Bio-Rad, Hercules, CA). Data were normalized to values for *ACT1* mRNA.

### Coimmunoprecipitation.

Cells expressing *Cg*Med2-Myc and *Cg*Yap6-His were grown to logarithmic phase in YNB medium. The cells were then cultured at pH 5.5 and pH 2.0 for 1 h and harvested at 4°C. Pellets were resuspended in lysis buffer (45 mM HEPES-KOH [pH 7.5], 150 mM NaCl, 1 mM EDTA, 10% glycerol, 1% Triton X-100, 2 mM dithiothreitol [DTT]) with protease and phosphatase inhibitors, followed by the addition of glass beads and sonication at 4°C. Protein extracts were clarified by centrifugation at 6,000 × *g* for 10 min at 4°C. Proteins were incubated with primary antibody at 4°C overnight and then incubated with protein A-agarose beads (Sangon Biotech) at 4°C for 6 to 8 h. Beads were washed six times with lysis buffer and one time with PBS and then boiled in SDS loading buffer for 10 min. The binding proteins were resolved by 10% SDS-PAGE and detected by Western blotting.

### Two-hybrid analysis.

The yeast two-hybrid assays were performed using a Matchmaker library construction and screening kit (Clontech), and two-hybrid analysis was carried out by using pGADT7 (Gal4AD) as the activation domain (AD) plasmid and pGBKT7 (Gal4BD) as the DNA-binding domain (BD) plasmid. The plasmid pGADT7-*Cg*Yap6 was cotransformed with pGBKT7-*Cg*Med2 using the AH109 reporter strain. Positive clones were selected and further tested as follows. The transformed yeast strains were grown until mid-log phase in YNB medium, diluted on synthetic dextrose (SD)-Leu-Trp plates and SD-Leu-Trp-His selective plates with the histidine biosynthesis inhibitor 1,2,4-aminotrizole (3-AT), and incubated for 2 to 4 days at 30°C.

### Alkaline phosphatase treatment.

Samples used for the alkaline phosphatase treatment were processed as described for Western blotting, except that after the lysis buffer washes, PBS was added to the immunoprecipitated proteins bound to the beads. Samples were treated with alkaline phosphatase (D7027; Beyotime) in the presence or absence of alkaline phosphatase inhibitor (P1081; Beyotime) and incubated for 1 h at 30°C with occasional shaking. Untreated samples were used as controls. The immunoprecipitated proteins were then washed twice with the wash buffer without protease inhibitors and released from the beads by boiling in SDS loading buffer.

### ChIP assay.

Cells were grown to the logarithmic phase and then cultured at pH 5.5 and pH 2.0 for 1 h. The cells were cross-linked with 1% formaldehyde for 20 min at room temperature. Glycine was added to a final concentration of 330 mM, and the incubation was continued for 15 min. The cells were collected, washed four times with cold Tris-buffered saline (TBS) (20 mM Tris-HCl [pH 7.5], 150 mM NaCl), and maintained at –20°C for further processing. Cell pellets were resuspended in 0.3 ml of cold lysis buffer (50 mM HEPES-KOH [pH 7.5], 150 mM NaCl, 1 mM EDTA, 0.1% sodium deoxycholate, 0.1% SDS, 1 mM phenylmethylsulfonyl fluoride [PMSF]) supplemented with 1% Triton X-100 and lysed with a bead beater. The cross-linked chromatin was sonicated to yield an average DNA fragment size of 350 bp (range, 100 to 850 bp). Finally, the sample was clarified by centrifugation at 12,000 × *g* for 5 min at 4°C. An aliquot of chromatin solution was used for immunoprecipitation (IP), input (IN), and control immunoprecipitation (CIP), and the remaining samples were stored at −20°C. The IP and CIP samples were incubated with anti-His monoclonal antibody and anti-IgG monoclonal antibody, respectively, which were precoupled to magnetic beads (9006; Cell Signaling Technology). After shaking for 2 h at 4°C on a rotator, the beads were washed twice with lysis buffer, twice again with lysis buffer plus 500 mM NaCl, twice with washing buffer (10 mM Tris-HCl [pH 8.0], 0.25 M LiCl, 1 mM EDTA, 0.5% N-P40, 0.5% sodium deoxycholate), and once with TE buffer (10 mM Tris-HCl [pH 8.0], 1 mM EDTA). The chromatin was eluted, and cross-linking was reversed by incubation at 65°C overnight. After extraction with phenol-chloroform-isoamyl alcohol (25:24:1, vol/vol/vol), DNA was ethanol precipitated for 4 h at −20°C and resuspended in TE buffer. qRT-PCR was used to analyze the DNA with TB Green Premix *Ex Taq* (TaKaRa Bio) on an iQ5 continuous fluorescence detector system (Bio-Rad, Hercules, CA, USA). Relative fold enrichment was calculated by using the following formula: fold change = (IP intensity – CIP intensity)/IN intensity. The primers used in this study are listed in Table 4.

### Fluorescence microscopy analysis.

Yeast strains carried the plasmids pY26-*Cg*Med2-eGFP and pY26-*Cg*Yap6-eGFP were cultivated in the logarithmic phase and then incubated at pH 2.0 for 1 h. Cells were then harvested and washed twice with 0.1 M phosphate buffer (PBS, pH 7.5). The pellet was resuspended in PBS at appropriate concentrations. Five microliters of suspension was put on a glass slide and images were obtained with a Leica TCS SP8 confocal microscope using 488 nm for eGFP. The percentage of cells with eGFP was calculated from three independent experiments and at least 100 cells per experiment at random.

### Glycerophospholipid extraction and measurement.

Logarithmic-phase C. glabrata cells were harvested and released into fresh YNB medium at pH 5.5 or 2.0 for 4 h. Cells were harvested, washed twice with PBS, and freeze-dried. Fifty milligrams of dried cells was resuspended in a solution of methanol, chloroform, and distilled water (1:2:1, vol/vol/vol). The sample extraction was carried out as described previously. The extracted glycerophospholipids were dried under a nitrogen stream and dissolved in methanol-isopropanol (1:1, vol/vol).

### Mass spectrometry analysis of glycerophospholipid.

Analysis of glycerophospholipid mixtures was carried out utilizing ultra-high-performance liquid chromatography–tandem mass spectrometry (UPLC-MS) (Waters, USA) and a CORTECS UPLC hydrophilic interaction liquid chromatography (HILIC) column (2.1 by 150 mm; inner diameter, 1.6 μm) with the gradient elution at 45°C and a rate of infusion of 0.3 ml · min^−1^. The mobile-phase gradient was formed by buffer A (acetonitrile) and buffer B (11 mM ammonium acetate). The A/B ratios were 95:5, 95:5, 70:30, 60:40, and 95:5 at run times of 0, 2, 15, 17, and 17.10 min, respectively. The capillary voltage was set at +3.5 kV or −3.5 kV for the positive or negative mode, respectively. Data analysis was based on the following commercial standards at a concentration of 1 mg · liter^−1^: 16:0 PA (830855; Avanti Polar Lipids), 16:0 PC (850355; Avanti Polar Lipids), 16:0 PS (840037; Avanti Polar Lipids), 16:0 PG (840455; Avanti Polar Lipids), 16:0 PE (850705; Avanti Polar Lipids), and 16:0 PI (850141; Avanti Polar Lipids). The mass amounts of glycerophospholipid were calculated by the following equation: content of glycerophospholipid = *a*_1_*c*_0_*v*/*a*_0_*m*, where *a*_1_ is the peak area of the sample, *a*_0_ is the peak area of the standard, *c*_0_ is the concentration of the standard, *v* is the total volume of the sample, and *m* is the mass of freeze-dried cells.

### Cell membrane integrity analysis.

Logarithmic-phase C. glabrata cells were harvested and released into fresh YNB medium at pH 5.5 or 2.0 for 4 h. Samples were centrifuged, washed twice with PBS, and diluted to an *A*_660_ of 0.5. The diluted sample (500 μl) was incubated with 5 μl of propidium iodide (Sangon Bio, Shanghai City, China) for 5 min at room temperature in the dark and then harvested, washed twice with PBS, and resuspended in the same volume of PBS. The cell number and fluorescence intensity (excitation, 536 nm; emission, 617 nm) of cell suspensions were measured by flow cytometry analysis using a FACSCalibur apparatus (BD Biosciences, Shanghai City, China). More than 20,000 events were analyzed for each sample and at a rate of 600 to 1,000 events/s. The data were acquired and analyzed using CellQuest software.

Cell membrane integrity was analyzed by confocal fluorescence microscopy. Logarithmic-phase C. glabrata cells were harvested and released onto fresh YNB medium at pH 5.5 or 2.0 for 4 h. The samples were centrifuged and washed twice with PBS (pH 7.4). The samples were then subjected to SYTOX green and FM4-64 uptake for 20 min and placed on a microscope slide covered with a coverslip. Images were acquired using a Nikon ECLIPSE 80i microscope equipped with a Nikon DS-Ri1 camera.

### Statistical analysis.

Experimental data are shown as the means ± standard deviations. All quantitative data were analyzed using Student's *t* test or one-way analysis of variance (ANOVA). Each experiment was repeated at least three times.

### Data availability.

The RNA-seq raw reads were submitted to NCBI under BioProject number PRJNA630869, and the Sequence Read Archive (SRA) entries are SRR11723916, SRR11723917, SRR11723918, and SRR11723919. The data that support the plots within this paper and other findings of this study are available from the corresponding author on reasonable request.

## Supplementary Material

Supplemental file 1

Supplemental file 2

Supplemental file 3

Supplemental file 4

Supplemental file 5
