# Supplementary material for: Candida glabrata Yap6 Recruits Med2 To Alter Glycerophospholipid Composition and Develop Acid pH Stress Resistance
Source: Appl Environ Microbiol. 2020 Nov 24;86(24):e01915-20. doi: 10.1128/AEM.01915-20 (PMC7688241; doi:10.1128/AEM.01915-20)
Supplement: Supplemental file 1 [file AEM.01915-20-s0001.pdf]

Supplementary Materials

Candida glabrata Yap6 recruits Med2 to alter glycerophospholipid composition and develop acid pH stress resistance

Pei Zhou[a,b,c], Xiaoke Yuan[d], Hui Liu[a,b,c], Yanli Qi[a,b,c], Liming Liu[a,b,c*]

[a]State Key Laboratory of Food Science and Technology, Jiangnan University, Wuxi, Jiangsu 214122, China

[b]Key Laboratory of Industrial Biotechnology, Ministry of Education, Jiangnan University, Wuxi 214122, China

[c]International Joint Laboratory on Food Safety, Jiangnan University, Wuxi 214122, China

[d]School of Biotechnology, Jiangnan University, Wuxi, Jiangsu 214122, China

[*]Address correspondence to:

Liming Liu

E-mail: mingll@jiangnan.edu.cn

Tel/Fax: +86-510-85197875

This file includes:

Supplementary Table 1 to 2

Supplementary Fig. S1 to S7

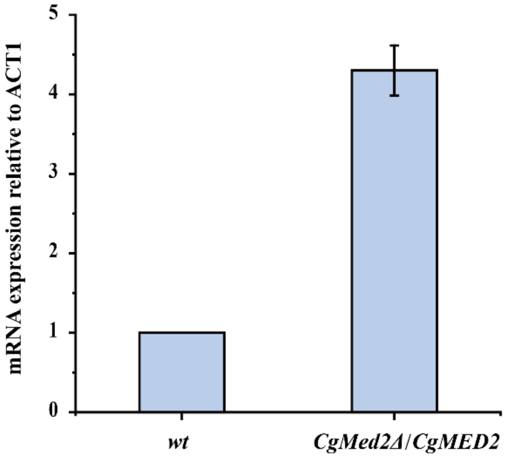

FIG S1 the expression level of genes Med2 in the wild-type (wt), and *Cg*Med2Δ/*Cg*MED2

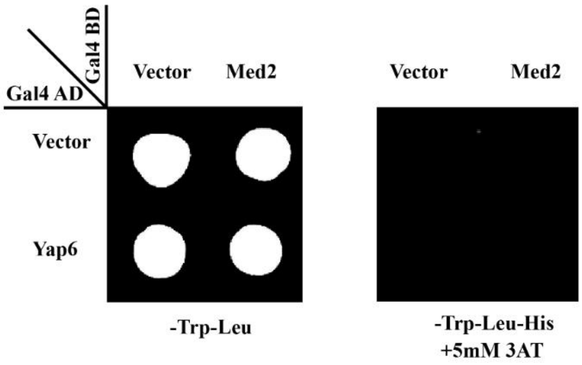

FIG S2 Yeast two-hybrid assay to detect the interaction between CgMed2 and CgYap6 at pH 5.5.

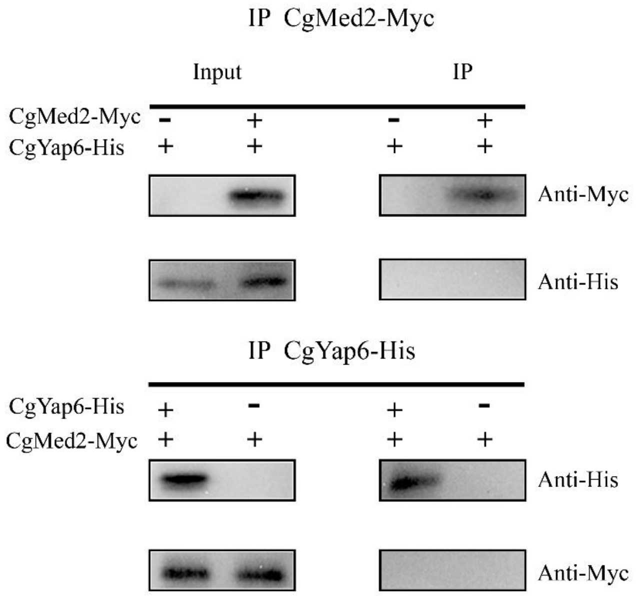

FIG S3 Co-immunoprecipitation assay to detect the interaction between CgMed2 and CgYap6 in vivo at pH 5.5.

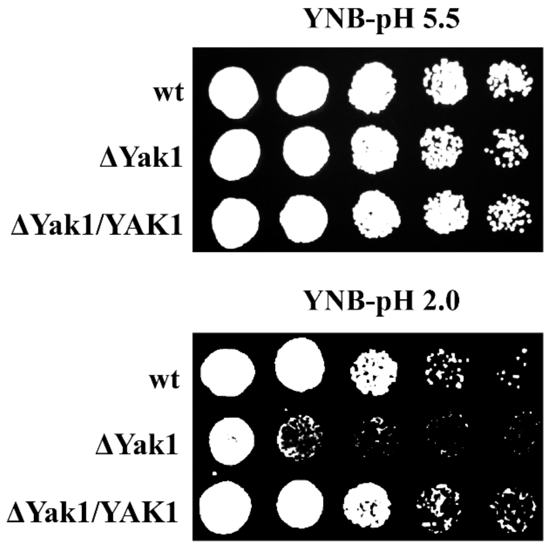

FIG S4 wild-type (wt), *Cg*Yak1Δ, and *Cg* Yak1Δ/*Cg*YAK1 were spotted on YNB plates at pH 5.5 and pH 2.0.

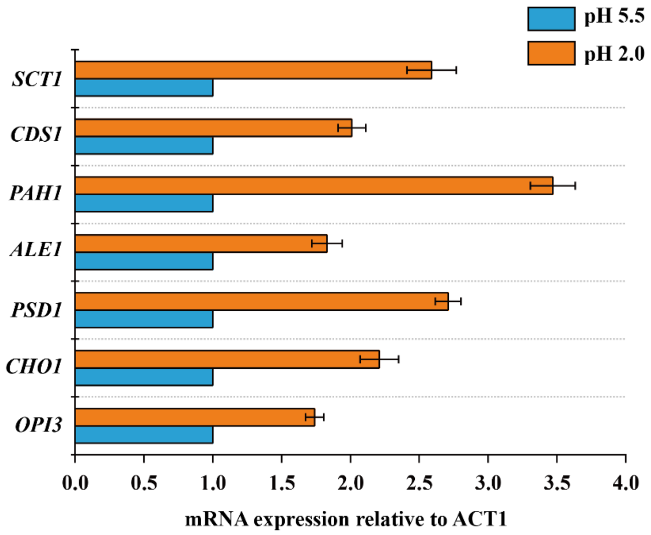

FIG S5 Quantitative reverse transcription-PCR (qRT-PCR) verified the wild-type (wt) mRNA expression levels of the glycerophospholipid genes, calculated relative to the ACT1 level, under pH 5.0 and pH 2.0 conditions.

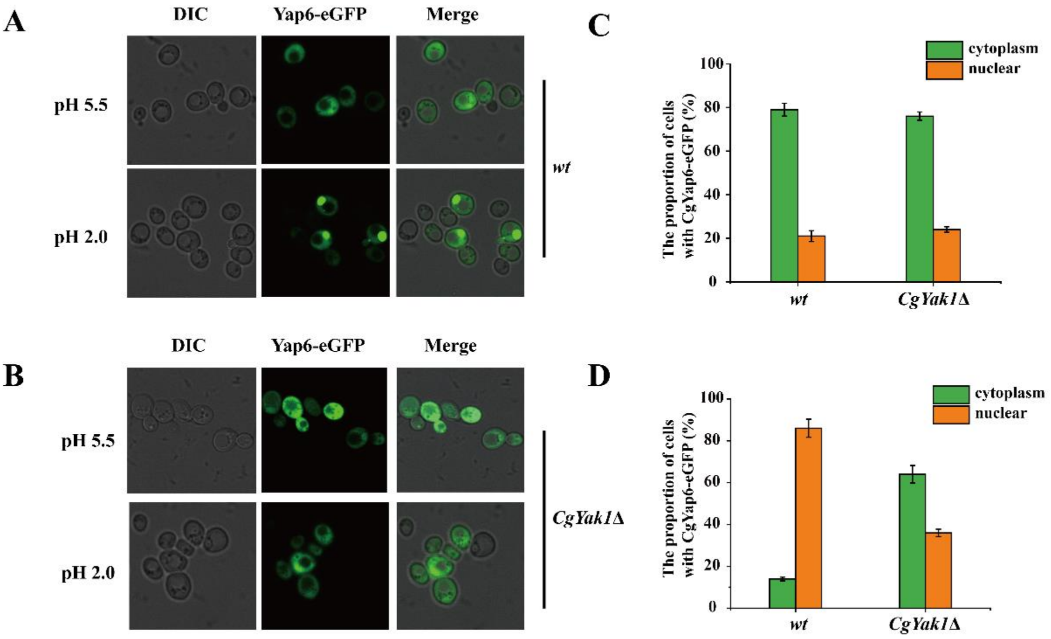

FIG S6 (A-B)The subcellular localization of CgYap6-GFP in the wild-type (wt) and CgYak1Δ strains at pH 5.5 and pH 2.0. (C-D) The quantification data of the subcellular location of CgYap6-

GFP in the wild-type (wt) and CgYak1Δ strains at pH 5.5 and pH 2.0.

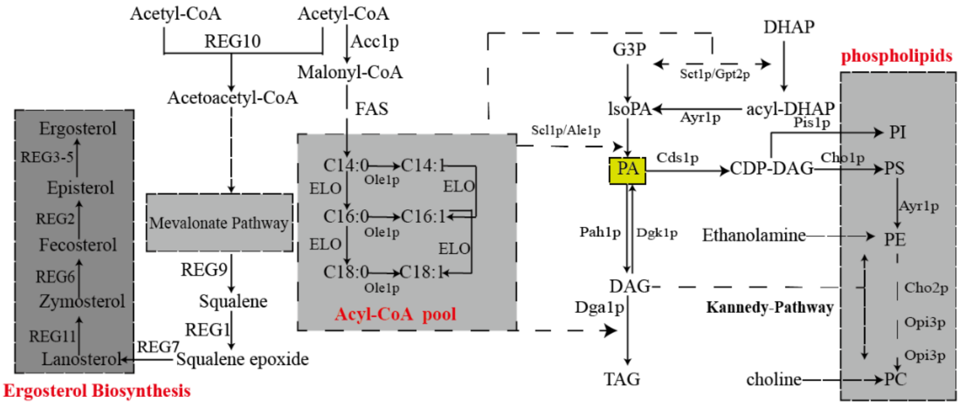

FIG S7 Lipid metabolic pathway of phospholipid synthesis in yeast. CDP-DAG pathway is the main phospholipid synthesis pathway, whereas Kennedy pathway is the compensation pathway.

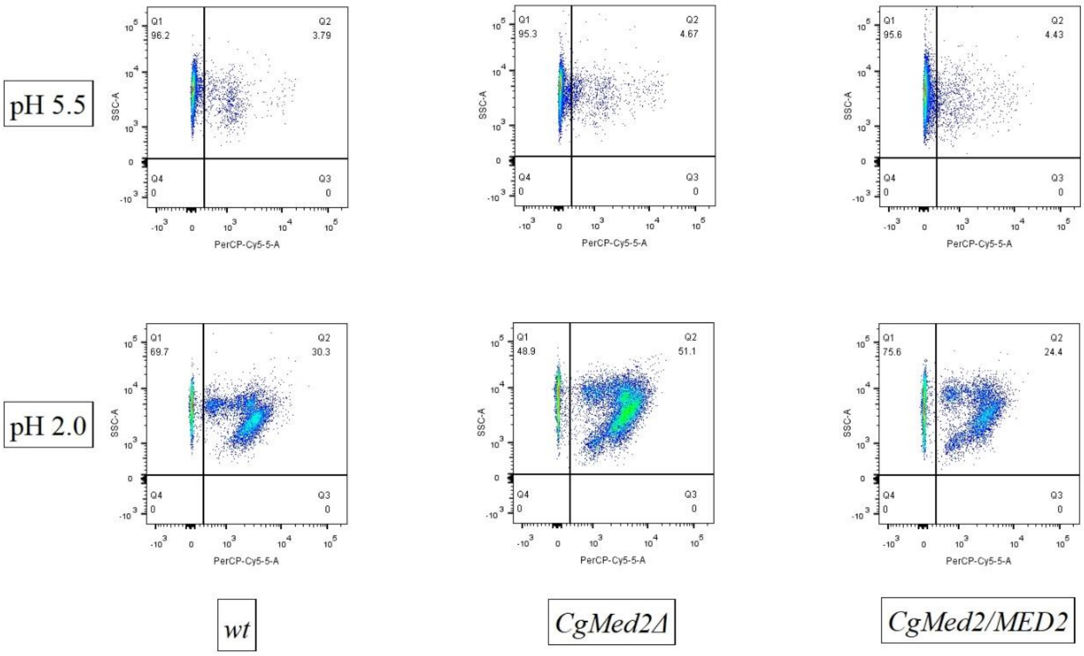

FIG S8 Flow cytometry analysis of membrane integrity in the wild-type (wt), *Cg*Med2Δ and *Cg*Med2Δ/*Cg*MED2 at pH 5.5 and pH 2.0.

Table S1: -logP of the wild-type strain and *Cg*Med2Δ at pH 5.5 and pH 2.0

**Pathway names**	**Part1**	**Part2**	**Part3**	**Part4**
Pyrimidine metabolism	6.8855	6.8014	0.96204	-
Propanoate metabolism	6.0628	-	6.0628	-
beta-Alanine metabolism	3.9245	2.3015	1.6094	-
Purine metabolism	3.3934	-	5.3014	0.64873
Inositol phosphate metabolism	3.3029	-	3.3029	-
Butanoate metabolism	3.2563	1.9646	3.2563	-
Galactose metabolism	3.2109	1.9415	1.2751	-
Fructose and mannose metabolism	2.9247	-	-	0.75295
Starch and sucrose metabolism	2.8515	1.7579	1.1089	2.7959
Citrate cycle (TCA cycle)	1.9165	0.76134	1.9165	0.68931
Alanine, aspartate and glutamate metabolism	1.7489	-	1.7489	-
Pantothenate and CoA biosynthesis	1.6421	2.3362	-	-
Valine, leucine and isoleucine biosynthesis	1.6421	-	1.6421	-
Phenylalanine metabolism	1.4904	2.1745	-	0.16524
Histidine metabolism	1.2153	-	1.2153	-
Ascorbate and aldarate metabolism	1.1964	1.855	-	-
Glycerolipid metabolism	1.1964	5.483	1.1964	2.8992
Glycine, serine and threonine metabolism	1.1426	1.7954	1.1426	-
Glyoxylate and dicarboxylate metabolism	1.1089	-	1.1089	0.25247
Pentose and glucuronate interconversions	1.0613	1.7045	-	-
Cysteine and methionine metabolism	1.0169	-	7.1487	-
Tyrosine metabolism	0.78156	-	0.78156	0.36581
Arginine and proline metabolism	0.77195	-	-	-
Amino sugar and nucleotide sugar metabolism	0.67622	5.6894	-	2.2466
Nitrogen metabolism	-	4.7591	-	3.0398
Aminoacyl-tRNA biosynthesis	-	3.5089	-	2.401
Tryptophan metabolism	-	1.3478	-	-
Sulfur metabolism	-	-	2.0145	-
Terpenoid backbone biosynthesis	-	-	1.4633	-
Glutathione metabolism	-	-	1.3403	0.47862
Phenylalanine, tyrosine and tryptophan biosynthesis	-	5.483	-	3.4025

Notes: Part 1: wt-pH 5.5 VS wt-pH 2.0; Part 2: wt-pH 2.0 VS CgMed2Δ-pH 2.0; Part 3: CgMed2Δ-pH 5.5 VS CgMed2Δ-pH 2.0; Part 4: wt-pH 5.5 VS CgMed2Δ-pH 5.5

Table S2: Transcriptome sequencing of sequencing data

Sample	Raw reads	Clean reads
**Wt-pH 2.0**	43293504	42242522
**Wt-pH 5.5**	44613436	43554258
***Cg*Med2Δ-pH 2.0**	45968744	44859046
***Cg*Med2Δ-pH 5.5**	44607820	43530178
